# Associations between body composition phenotypes and physical function in community-dwelling older adults: an exploratory analysis of limb muscle mass asymmetry

**DOI:** 10.3389/fmed.2026.1862786

**Published:** 2026-07-17

**Authors:** Yawen Lv, Ziwen Zhou, Xiaoyue Shen, Hongyu Wang

**Affiliations:** 1 Graduate School, Bengbu Medical University, Bengbu, China; 2 School of Humanities and Health, Bengbu Medical University, Bengbu, China

**Keywords:** body composition, cross-sectional study, latent profile analysis, muscle asymmetry, older adults, physical function

## Abstract

**Objective:**

To identify body composition phenotypes and compare physical function across phenotypes among community-dwelling older adults, with a secondary exploratory analysis of limb muscle mass asymmetry.

**Methods:**

This cross-sectional study included 600 community-dwelling adults aged 60–79 years in Bengbu, Anhui, China. Body composition was assessed using bioelectrical impedance analysis. Body fat percentage, trunk fat mass, and lower-limb muscle mass were standardized within sex and entered into latent profile analysis to identify body composition phenotypes. Upper- and lower-limb muscle mass asymmetry indices (AIs) were calculated and standardized for secondary exploratory analyses. Physical function was assessed using the 30-s chair stand, one-leg stance with eyes closed, grip strength, and 2-min high-knee stepping in place. ANCOVA, linear regression, logistic regression, subgroup analyses, and extreme-group analyses were performed, with FDR correction applied to regression-related analyses.

**Results:**

Three distinct body composition phenotypes were identified: low-adiposity phenotype (C1, 27%), balanced phenotype (C2, 55%), and high-adiposity–high-muscle phenotype (C3, 18%). After adjustment for age and sex, significant differences across phenotypes were observed in grip strength (*P* = 0.003), one-leg stance with eyes closed (*P* = 0.002), and 30-s chair stand performance (*P* = 0.003), but not in 2-min high-knee stepping in place. The high-adiposity–high-muscle phenotype had the highest grip strength, whereas the low-adiposity phenotype had the longest one-leg stance time and the best 30-s chair stand performance. Linear regression analyses showed no significant associations between upper- or lower-limb AI and continuous functional performance outcomes after FDR correction. In logistic regression analyses, upper-limb AI was nominally associated with sample-defined low one-leg stance performance (OR = 1.215, 95% CI: 1.017–1.452, *P* = 0.032), but this association did not remain significant after FDR correction. No significant association was observed for lower-limb AI.

**Conclusion:**

Community-dwelling older adults showed heterogeneous body composition phenotypes with significant differences in several physical function domains. Limb muscle mass asymmetry was not robustly associated with functional performance after correction for multiple comparisons. These findings suggest that body composition phenotypes may provide useful information for understanding functional heterogeneity in older adults, whereas the role of limb muscle mass asymmetry requires further investigation.

## 1 Introduction

Global population aging is widely recognized as one of the most profound demographic and socioeconomic transformations of the twenty-first century (1). With increasing life expectancy and declining fertility rates, both the number and proportion of older adults have continued to rise in almost all countries, driving a marked shift toward an aging population structure worldwide (2). By 2050, the global population aged 60 years and over is projected to reach more than 2.1 billion, accounting for over 21% of the total population (3). In this context, promoting healthy aging has become a major global public health priority, and maintaining good functional ability is considered a key component of healthy aging (4). Previous studies have shown that functional decline is closely associated with falls, frailty, and other geriatric syndromes, and can substantially impair older adults’ ability to perform activities of daily living and goal-directed behaviors (5). Therefore, the assessment of functional status is of great importance for disease prevention, early identification, and health management in older adults (6). As one of the countries experiencing the most rapid population aging, China has seen a substantial increase in its older population, making it particularly important to identify modifiable factors associated with functional health (7).

Among the potentially modifiable factors related to functional health, body composition has received increasing attention because of its important implications for physical function and metabolic status (8, 9). Previous studies have shown that loss of muscle mass can lead to reduced muscle strength, lower basal metabolic rate, and impaired aerobic capacity, thereby compromising overall physical function (8, 10). In contrast, excessive fat accumulation is closely associated with reduced physical capacity, increased risk of disability, and poorer quality of life (11, 12). Traditional studies have often relied on simple indicators, such as body mass index (BMI) or waist circumference, to assess body composition; however, these measures cannot distinguish muscle mass from fat mass and therefore fail to accurately reflect true changes in body composition (13, 14). In addition, body composition among older adults shows substantial heterogeneity, and a single indicator cannot fully capture the multidimensional distribution of fat and muscle. Therefore, person-centered analytical approaches, such as latent profile analysis (LPA), have gradually been applied to identify distinct body composition phenotypes (15).

Although previous studies have reported associations between body composition and physical function (16, 17), most have still relied on one or only a few simple indicators, making it difficult to capture the multidimensional characteristics of body composition (18, 19). In recent years, several studies have begun to explore functional differences across body composition phenotypes (20, 21); however, the available evidence remains limited and fragmented, and no consistent conclusions have yet been reached. Meanwhile, limb muscle asymmetry is common among older adults, and differences in muscle mass between the two sides may disrupt the distribution of muscle force, impair balance control and postural stability, and thereby increase the risk of functional decline and falls (22, 23). However, quantitative studies examining the association between the asymmetry index (AI) and physical function remain limited, particularly with respect to grip strength and balance function. In addition, existing evidence has lacked subgroup analyses and robustness checks. More importantly, few studies have incorporated body composition phenotypes, muscle asymmetry, and physical function into a unified analytical framework. Empirical evidence is still lacking regarding whether different body composition phenotypes may modify the association between muscle asymmetry and physical function. Therefore, integrating these factors in a single study is important for understanding the mechanisms underlying functional decline in older adults and for informing individualized interventions.

Based on the above research gaps, this study was conducted among a sample of community-dwelling older adults aged 60–79 years in Bengbu City in 2025. First, LPA was used to identify latent classes based on body fat percentage, trunk fat mass, and lower-limb muscle mass, and differences in physical function indicators, including grip strength and balance performance, were compared across phenotypes. Second, the AI of upper- and lower-limb muscle mass was calculated to examine its associations with grip strength, balance function, and risk of low balance performance and low grip strength, as well as the potential modifying role of body composition phenotypes in these associations. Finally, sex-stratified, age-stratified, and extreme-value analyses were conducted to assess the robustness of the findings. This study aimed to examine the associations of body composition heterogeneity and muscle asymmetry with physical function in older adults, and to provide locally relevant evidence for the early identification of functional heterogeneity and the development of individualized interventions.

## 2 Materials and methods

### 2.1 Study design and participants

This was a cross-sectional study conducted in Bengbu, Anhui, China. Community-dwelling older adults aged 60–79 years were recruited from urban and rural areas between July and August 2025 using stratified random sampling. Data were collected by trained investigators using standardized procedures. A total of 608 participants were initially recruited. During data quality checking, three functional test records were judged invalid or unreliable because their values were implausibly low and inconsistent with valid completion of the standardized physical fitness tests. These records were not treated as reliable true extreme observations. After excluding these 3 participants and 5 participants with missing key variables, 600 participants were included in the final analysis.

Eligible participants were community-dwelling older adults aged 60–79 years who were able to understand and complete the physical fitness tests. Exclusion criteria included severe cardiocerebrovascular or metabolic diseases, motor or cognitive impairment that could affect completion of the tests, and any health condition on the day of testing that made participation inappropriate.

The surveillance covered three categories of indicators: body morphology, physiological function, and physical fitness. Anthropometric measurements included height, weight, waist circumference, hip circumference, and body fat percentage. Physiological function indicators included vital capacity, heart rate, systolic blood pressure, and diastolic blood pressure. Physical fitness tests included grip strength (kg), 2-min high-knee stepping in place (steps), sit-and-reach (cm), 30-s chair stand (reps), one-leg stance with eyes closed (s), and choice reaction time (s). The study was approved by the Ethics Committee of Bengbu Medical University (approval No. 2025-034), and written informed consent was obtained from all participants before participation.

### 2.2 Measurements

#### 2.2.1 Body composition measurements

Body composition was assessed using a body composition analyzer (InBody770C, Shanghai Baisen Medical Technology Co., Ltd., China). The device estimates body composition based on bioelectrical impedance analysis (BIA).

All measurements were performed during daytime. Participants wore light clothing and were tested in a fasting state or at least 2 h after a meal. Before measurement, they rested in a seated position for 5 min and removed all metal accessories.

Body fat percentage (FAT/W, %), trunk fat mass (kg), and lower-limb muscle mass (kg) were selected as body composition indicators for LPA. The selection of these indicators was guided by conceptual relevance and redundancy reduction. Before LPA, pairwise Pearson correlations were examined among the BIA-derived body composition variables considered for profile construction. Variables with high statistical redundancy (|r| > 0.85) or clear conceptual overlap were not entered into the same LPA model.

Body fat percentage was retained to represent overall adiposity because it directly reflects the relative proportion of body fat and is more informative than BMI, which cannot distinguish fat mass from muscle mass. Trunk fat mass was retained to represent central fat accumulation, whereas waist circumference was not entered into the LPA because it is an indirect anthropometric indicator and partly overlaps with central fat distribution. Lower-limb muscle mass was retained to represent functionally relevant muscle reserve because several outcomes in this study, including one-leg stance with eyes closed, 30-s chair stand, and 2-min high-knee stepping in place, are closely related to lower-limb function.

Whole-body skeletal muscle mass and fat-free mass were not selected as LPA indicators because they are broader whole-body summary measures and partly overlap with appendicular muscle reserve and overall body size. Body fat mass was also not selected because it is an absolute total fat measure that overlaps with body fat percentage and body size. Therefore, the final three indicators captured three complementary and functionally relevant dimensions of body composition: overall adiposity, central fat accumulation, and lower-limb muscle reserve. Lower-limb muscle mass was calculated as the mean of the left and right lower-limb muscle mass values. LPA was then applied to identify body composition phenotypes.

#### 2.2.2 Functional performance

Physical function was evaluated using four tests: 30-s chair stand (reps), 2-min high-knee stepping in place (steps), grip strength (kg), and one-leg stance with eyes closed (s).

For the 30-s chair stand (reps), participants sat on a chair with a seat height of 43 cm, with both arms crossed over the chest and both feet flat on the floor. After the start signal, they repeatedly stood up and sat down as quickly as possible for 30 s. The number of completed stands was recorded.

For the 2-min high-knee stepping in place (steps), participants stood in place and swung both arms naturally while alternately lifting the knees until the thighs were parallel to the floor, with approximately 90° of knee flexion. The test lasted 2 min, and the number of valid steps was recorded.

Grip strength (kg) was measured bilaterally using a Jianmin electronic hand dynamometer (model GMCS-WCS3, Beijing Xindonghuateng Sports Equipment Co., Ltd., China). Participants stood upright with both feet naturally apart and the tested arm hanging naturally at the side. Each hand was tested twice, and the maximum value for each side was recorded. The higher of the two maximum values was used for analysis.

For the one-leg stance with eyes closed (s), participants stood on one leg on a flat surface with both hands on the waist and the eyes closed, while the other foot was lifted off the ground. In the available analytic dataset, only one final recorded value for this test was retained, and side-specific results for the left and right legs were not available. Therefore, this variable was used as an overall indicator of static balance performance rather than as a measure of side-specific balance asymmetry.

#### 2.2.3 Covariates and descriptive variables

Age and sex were included as covariates in the adjusted functional performance and regression models. Urban-rural residence, BMI, waist circumference, skeletal muscle mass, fat-free mass, and body fat mass were used for descriptive comparisons across body composition phenotypes.

### 2.3 Asymmetry index

To assess bilateral differences in upper- and lower-limb muscle mass in older adults, the AI was calculated to quantify side-to-side asymmetry in limb muscle mass. The upper-limb AI was derived from the muscle mass of the left and right upper limbs, and the lower-limb AI was derived from the muscle mass of the left and right lower limbs.

The absolute asymmetry index was calculated as follows:


$$A\,I=\frac{\left|L-R\right|}{\text{L}+\text{R}}\times 100\%$$

where L and R denote the muscle mass (kg) of the left and right limbs, respectively. The AI ranges from 0% to 100%, with higher values indicating greater bilateral asymmetry. This method has been widely used to evaluate asymmetry in bilateral limb muscle mass or functional performance (24, 25).

For subsequent statistical analyses, the upper- and lower-limb AI values were standardized using Z-scores to reduce the influence of differences in measurement scale and to improve comparability across indicators.

### 2.4 Latent profile analysis

Latent profile analysis was used to identify latent subgroups based on body composition characteristics. LPA models with continuous indicators were estimated using Mplus software (Version 8.3). The observed variables included body fat percentage (FAT/W, %), trunk fat mass (kg), and lower-limb muscle mass (kg). These indicators were standardized within sex before being entered into the model as continuous variables.

Model fitting began with a one-class model, and the number of latent classes was increased sequentially. Model fit was evaluated using multiple indices, including the Akaike Information Criterion (AIC), Bayesian Information Criterion (BIC), adjusted Bayesian Information Criterion (aBIC), entropy, the Lo-Mendell-Rubin likelihood ratio test (LMR-LRT), and the bootstrap likelihood ratio test (BLRT). Lower AIC, BIC, and aBIC values indicate better model fit, whereas entropy values closer to 1 indicate higher classification accuracy. The LMR-LRT and BLRT were used to compare models with adjacent numbers of classes, and a *P*-value < 0.05 indicated that the addition of one more class significantly improved model fit.

The final number of classes was determined based on an overall consideration of model fit indices, the plausibility of class proportions, and the clinical interpretability and practical relevance of the classification results. Solutions with overly small classes were avoided.

### 2.5 Comparison across classes

Demographic characteristics, anthropometric indicators, body composition indicators, and physical function were compared across latent classes. One-way ANOVA or Welch’s ANOVA was used for continuous variables according to Levene’s test, and the chi-square test was used for categorical variables. Differences in physical function indicators across latent classes were further evaluated using ANCOVA adjusted for age and sex. Adjusted means, that is, estimated marginal means, were calculated for each class.

### 2.6 Association analyses

To examine the associations between limb muscle mass asymmetry indices and physical function, linear regression models were first fitted for each continuous functional outcome. Functional indicators were treated as dependent variables, and upper- or lower-limb AI was treated as the independent variable. Asymmetry indices were standardized before regression analyses, and all models were adjusted for age and sex.

To evaluate whether body composition phenotypes modified the association between AI and physical function, an interaction term between AI and latent class membership (AI × class) was included in the models.

Logistic regression analyses were further conducted to examine the associations between AI and the risk of low functional performance. Low functional performance was defined as values at or below the sample-specific first quartile of each functional indicator. This definition reflected relatively low performance within the present study sample rather than a clinically established impairment threshold. Because only one final recorded value for the one-leg stance test was retained in the available analytic dataset, this outcome was analyzed only as a general indicator of static balance performance. Therefore, weaker-side performance, bilateral average performance, and side-to-side balance differences could not be examined. These models were also adjusted for age and sex. To further assess the robustness of the findings, subgroup analyses were conducted according to sex and age group, and extreme-group analyses were performed by comparing participants in the highest and lowest 10% of the upper- or lower-limb AI distribution.

### 2.7 Statistical analysis

All statistical analyses were performed using Python (Version 3.12.4) and Mplus (Version 8.3). Before LPA, body fat percentage, trunk fat mass, and lower-limb muscle mass were standardized within sex to reduce the influence of sex-related differences in body composition. LPA was conducted in Mplus using these sex-standardized indicators as continuous variables. Model fit was evaluated using the AIC, BIC, aBIC, entropy, the LMR-LRT, and the BLRT. The final number of classes was determined based on model fit indices, class proportions, and clinical interpretability.

Differences across classes were examined using one-way ANOVA or Welch’s ANOVA for continuous variables according to Levene’s test, and the chi-square test for categorical variables. Differences in functional performance across classes were further evaluated using ANCOVA adjusted for age and sex. Linear regression and logistic regression analyses were used to evaluate the associations between limb muscle mass AI and continuous or low functional performance outcomes. All regression models were adjusted for age and sex. Interaction, subgroup, and extreme-group analyses were further performed to assess the robustness of the findings. FDR correction was applied using the Benjamini–Hochberg procedure for regression-related analyses, including linear regression, logistic regression, interaction, subgroup, and extreme-group analyses. Raw *P*-values and FDR-adjusted *P*-values were reported where applicable. Raw *P* < 0.05 was considered nominally significant, whereas FDR-adjusted *P* < 0.05 was considered significant after correction for multiple comparisons. All tests were two-sided.

## 3 Results

### 3.1 Participant characteristics

A total of 608 older adults aged 60–79 years were initially recruited. After excluding 3 participants with invalid or unreliable functional test records, as defined in the section “2 Materials and methods,” and 5 participants with missing key variables, 600 participants were included in the final analytic sample, including 231 men (38.5%) and 369 women (61.5%). The mean age was 67.78 ± 5.00 years. The mean BMI was 24.43 ± 3.11 kg/m^2^, and the mean waist circumference was 86.02 ± 11.01 cm. Descriptive statistics for other body composition and functional indicators are presented in Supplementary Table 1.

Based on sex-standardized latent profile analysis (LPA), participants were classified into three latent classes, which were interpreted as body composition phenotypes: C1 (low-adiposity phenotype; *n* = 164), C2 (balanced phenotype; *n* = 332), and C3 (high-adiposity–high-muscle phenotype; *n* = 104). No significant differences were observed among the three classes in demographic characteristics, whereas significant differences were found in anthropometric and body composition indicators.

In terms of demographic characteristics, the three classes were comparable in age, sex distribution, and urban-rural distribution, with no significant between-class differences observed (all *P* > 0.05; Table 1).

**TABLE 1:** Baseline characteristics of participants across body composition phenotypes.

Variable	C1 (*n* = 164)	C2 (*n* = 332)	C3 (*n* = 104)	Statistic	*p*-value
Demographics					
Age, years	68.3 ± 5.0	67.6 ± 5.0	67.5 ± 4.9	1.25	0.287
Sex, *n* (%)	100 (61.0%)	206 (62.0%)	63 (60.6%)	0.10	0.952
Female					
Male	64 (39.0%)	126 (38.0%)	41 (39.4%)		
Residence (urban/rural), *n* (%)				1.03	0.598
Urban	128 (78.0%)	270 (81.3%)	81 (77.9%)		
Rural	36 (22.0%)	62 (18.7%)	23 (22.1%)		
Anthropometrics					
Body mass index (BMI), kg/m^2^	21.2 ± 1.8	24.7 ± 1.8	28.6 ± 2.4	417.80	<0.001
Waist circumference, cm	77.9 ± 7.3	87.0 ± 10.6	95.8 ± 7.1	122.86	<0.001
Body Composition					
Whole-body muscle mass, kg	38.8 ± 6.8	41.5 ± 7.1	45.9 ± 7.6	31.78	<0.001
Fat-free mass, kg	41.3 ± 7.2	44.2 ± 7.5	48.9 ± 8.1	31.99	<0.001
Fat mass, kg	13.4 ± 3.0	20.3 ± 2.7	28.0 ± 3.3	700.97	<0.001

Significant differences were observed among the three classes in BMI, waist circumference, whole-body muscle mass, fat-free mass, and body fat mass (all *P* < 0.001). Across these indicators, C3 consistently showed the highest values, C1 showed the lowest values, and C2 showed intermediate values. Detailed values and group comparisons are shown in Table 1.

### 3.2 LPA results

Latent profile analysis models were fitted sequentially from one to five classes using sex-standardized body composition indicators, and the model fit indices are shown in Table 2. As the number of classes increased, the AIC, BIC, and aBIC values decreased. The LMR-LRT and BLRT results showed that the three-class model provided a significantly better fit than the two-class model (LMR-LRT *P* = 0.0012; BLRT *P* < 0.001), with acceptable classification accuracy (entropy = 0.856). Although the information criteria continued to decrease in the four- and five-class models, the four-class model did not significantly improve model fit compared with the three-class model (LMR-LRT *P* = 0.0698). Although the five-class model showed a significant LMR-LRT result, the two smallest classes accounted for only 10% and 8% of the sample and mainly represented further subdivisions of the existing phenotypes rather than clearly distinct and clinically meaningful patterns. In addition, models with more classes increased model complexity and did not provide substantially clearer clinical interpretability. Therefore, considering model fit, parsimony, class proportions, and phenotypic interpretability, the three-class model was selected as the optimal solution.

**TABLE 2:** Model fit indices for sex-standardized latent profile analysis models.

Classes	AIC	BIC	aBIC	LMR (P)	BLRT (P)	Entropy	Class proportions
1	5188.288	5214.749	5195.700	–	–	–	1
2	4720.639	4764.741	4732.993	0.0041	<0.001	0.740	0.42/0.58
3	4307.965	4369.707	4325.261	0.0012	<0.001	0.856	0.27/0.55/0.18
4	4118.105	4197.488	4140.343	0.0698	<0.001	0.866	0.30/0.13/0.12/0.45
5	3960.811	4057.834	3987.99	0.0181	<0.001	0.879	0.40/0.24/0.10/0.08/0.18

The proportions of the three latent classes were approximately 27.0% for C1 (low-adiposity phenotype), 55.0% for C2 (balanced phenotype), and 18.0% for C3 (high-adiposity–high-muscle phenotype).

**FIGURE 1:**
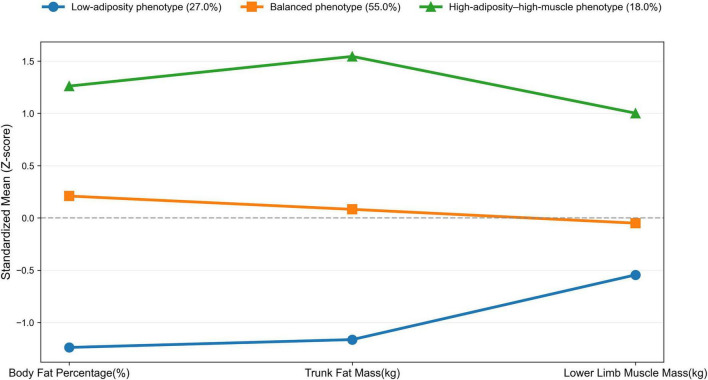
Sex-standardized mean profiles of the three latent classes.

Clear differences in sex-standardized body composition indicators were observed across the three classes (Figure 1). C1 was characterized by markedly lower body fat percentage and trunk fat mass, with mildly lower lower-limb muscle mass. C2 showed values close to the sex-specific average across the three indicators, representing a balanced body composition pattern. C3 showed higher body fat percentage, trunk fat mass, and lower-limb muscle mass, indicating a high-adiposity–high-muscle pattern. Overall, the three latent phenotypes showed distinct patterns in the relative distribution of adiposity and muscle mass.

### 3.3 Functional differences across body composition phenotypes

After adjustment for age and sex, ANCOVA showed significant between-group differences in some functional indicators across body composition phenotypes (Table 3).

**TABLE 3:** Adjusted functional performance across body composition phenotypes.

Outcome	C1 (*n* = 164)	C2 (*n* = 332)	C3 (*n* = 104)	F	*P*-value	η ^2^
30-s chair stand (reps)	17.54 ± 0.35	16.77 ± 0.25	15.60 ± 0.44	5.88	0.003	0.019
One-leg stance with eyes closed (s)	10.14 ± 0.49	7.98 ± 0.34	8.89 ± 0.61	6.57	0.002	0.022
Grip strength (kg)	22.44 ± 0.44	23.73 ± 0.31	24.81 ± 0.56	5.89	0.003	0.019
2-min high-knee stepping in place (steps)	94.63 ± 2.38	94.49 ± 1.67	97.56 ± 2.98	0.43	0.654	0.001

**FIGURE 2:**
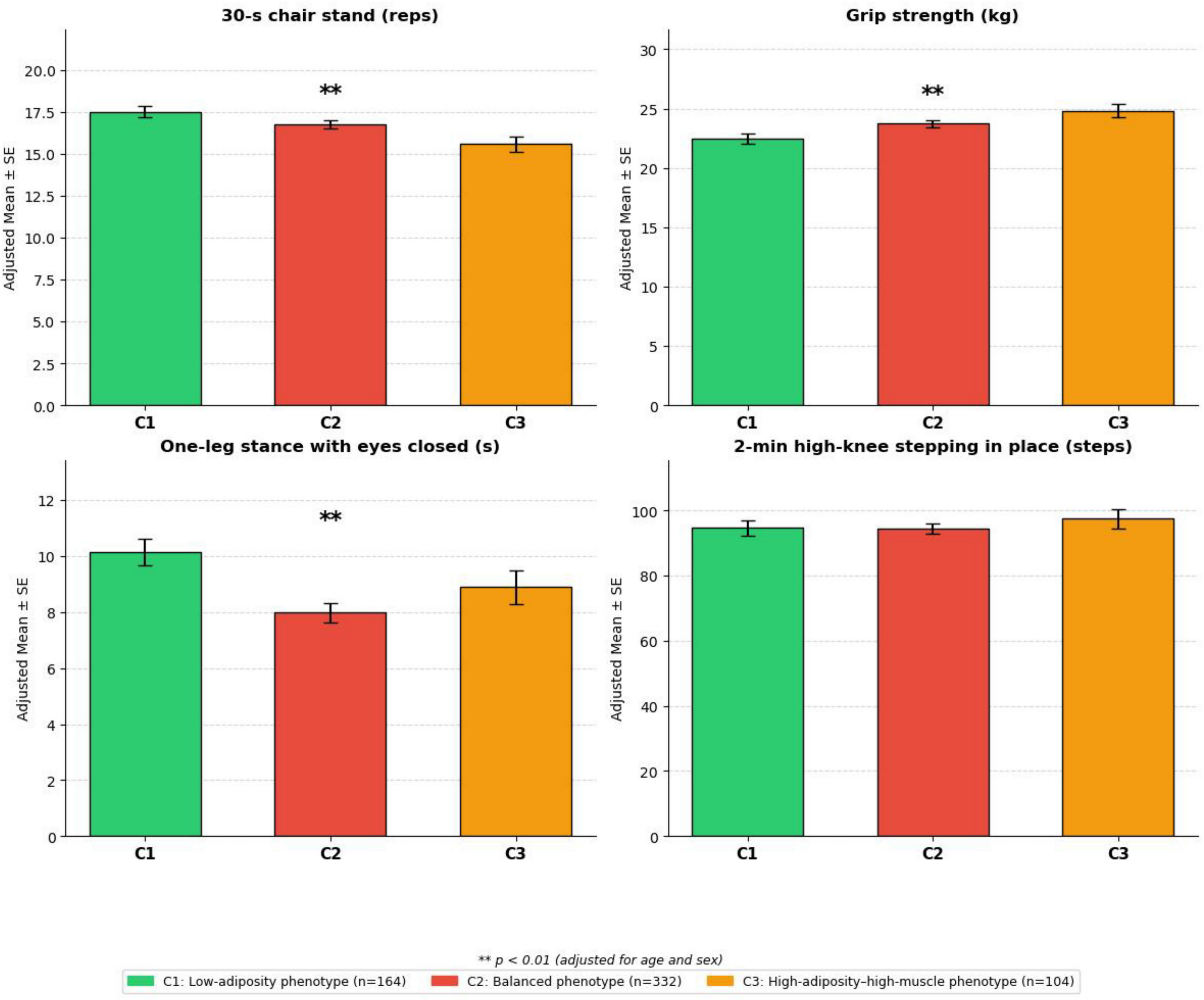
Adjusted functional performance across body composition phenotypes.

Grip strength differed significantly across phenotypes (*F* = 5.89, *P* = 0.003). The adjusted means showed that C3 (High-adiposity–high-muscle phenotype) had the highest grip strength (24.81 ± 0.56 kg), followed by C2 (Balanced phenotype; 23.73 ± 0.31 kg), whereas C1 (Low-adiposity phenotype) had the lowest value (22.44 ± 0.44 kg) (Figure 2). One-leg stance with eyes closed also differed significantly across phenotypes (*F* = 6.57, *P* = 0.002). C1 had the longest standing time (10.14 ± 0.49 s), followed by C3 (8.89 ± 0.61 s), whereas C2 had the shortest time (7.98 ± 0.34 s) (Figure 2). The 30-s chair stand also showed significant between-group differences (*F* = 5.88, *P* = 0.003), with C1 showing the highest adjusted mean (17.54 ± 0.35 reps), followed by C2 (16.77 ± 0.25 reps) and C3 (15.60 ± 0.44 reps) (Figure 2).

No significant between-group difference was observed for the 2-min high-knee stepping in place (*F* = 0.43, *P* = 0.654). The unadjusted mean comparisons are presented in Supplementary Table 2. Although significant differences were observed in grip strength, one-leg stance with eyes closed, and 30-s chair stand performance, the corresponding effect sizes were small (η^2^ = 0.019–0.022). Therefore, these findings indicate modest group-level functional differences across body composition phenotypes.

### 3.4 Linear associations between asymmetry and functional performance

After adjustment for age and sex, neither upper-limb nor lower-limb AI was significantly associated with any continuous functional performance outcome, including 30-s chair stand, one-leg stance with eyes closed, grip strength, and 2-min high-knee stepping in place. All associations remained non-significant after FDR correction (Supplementary Table 3).

No significant interactions were observed between limb AI and body composition phenotype for any continuous functional performance outcome after adjustment for age and sex. These interaction effects also remained non-significant after FDR correction (Supplementary Table 4).

### 3.5 Logistic regression analysis of asymmetry and low functional performance

After adjustment for age and sex, logistic regression analyses showed that upper-limb AI was nominally associated with low one-leg stance performance (OR = 1.215, 95% CI: 1.017–1.452, *P* = 0.032). However, this association did not remain significant after FDR correction (FDR-adjusted *P* = 0.257).

Upper-limb AI was not significantly associated with low 30-s chair stand, low grip strength, or low 2-min high-knee stepping performance. Lower-limb AI was not significantly associated with any low functional performance outcome. Detailed results are presented in Table 4.

**TABLE 4:** Logistic regression analysis of limb muscle mass asymmetry indices and low functional performance.

Predictor	Low-performance outcome	OR (95% CI)	*P*-value	FDR-adjusted *P*
Upper limb AI	Low 30-s chair stand	0.960 (0.798, 1.156)	0.668	0.891
	Low one-leg stance with eyes closed	1.215 (1.017, 1.452)	0.032	0.257
	Low grip strength	1.129 (0.924, 1.380)	0.236	0.630
	Low 2-min high-knee stepping	0.992 (0.825, 1.193)	0.929	0.932
Lower limb AI	Low 30-s chair stand	0.992 (0.815, 1.206)	0.932	0.932
	Low one-leg stance with eyes closed	0.946 (0.768, 1.166)	0.605	0.891
	Low grip strength	0.930 (0.748, 1.156)	0.514	0.891
	Low 2-min high-knee stepping	1.118 (0.943, 1.326)	0.199	0.630

Low functional performance was defined as values at or below the sample-specific first quartile and indicates relatively low performance within this sample, not a clinical impairment threshold. Models were adjusted for age and sex. ORs are presented per 1-SD increase in the asymmetry index. FDR-adjusted *P*-values were calculated using the Benjamini–Hochberg procedure.

### 3.6 Stratified and extreme-group analyses

Stratified analyses were performed to further evaluate the association between upper-limb AI and low balance performance (Table 5). In sex-stratified analyses, upper-limb AI was nominally associated with low balance performance in women (OR = 1.345, 95% CI: 1.040–1.738, *P* = 0.024), but this association did not remain significant after FDR correction (FDR-adjusted *P* = 0.074). No significant association was observed in men.

**TABLE 5:** Stratified analysis of upper-limb asymmetry and low balance performance.

Subgroup	OR (95% CI)	*P*-value	FDR-adjusted *P*
Sex			
Male	1.116 (0.869, 1.434)	0.389	0.486
Female	1.345 (1.040, 1.738)	0.024	0.074
Age group			
≤75 years	1.225 (1.020, 1.471)	0.030	0.074
>75 years	1.247 (0.574, 2.709)	0.578	0.578

Low balance performance was defined as one-leg stance with eyes closed ≤5.0 s, corresponding to the sample-specific first quartile rather than a clinical impairment threshold. ORs were adjusted for age or sex as appropriate. FDR-adjusted *P*-values were calculated using the Benjamini–Hochberg method.

In age-stratified analyses, upper-limb AI was nominally associated with low balance performance among participants aged ≤75 years (OR = 1.225, 95% CI: 1.020–1.471, *P* = 0.030), but this association did not remain significant after FDR correction (FDR-adjusted *P* = 0.074). No significant association was observed among participants aged >75 years. The interaction terms for sex and age group were not significant.

In the extreme-group analysis, participants in the highest 10% of upper-limb AI had nominally higher odds of sample-defined low balance performance than those in the lowest 10% group (OR = 1.870, 95% CI: 1.021–3.426, *P* = 0.043), but this association did not remain significant after FDR correction (FDR-adjusted *P* = 0.085). No significant association was observed for lower-limb AI (Supplementary Table 5).

## 4 Discussion

Based on a sample of community-dwelling older adults, this study identified three distinct body composition phenotypes and found significant differences in several functional performance indicators across phenotypes. In contrast, limb muscle mass asymmetry was not robustly associated with functional performance after correction for multiple comparisons. Upper-limb AI showed only a nominal association with low one-leg stance performance, and this association did not remain significant after FDR correction. These findings suggest that functional status in older adults may be related to body composition patterns, whereas the association between limb muscle mass asymmetry and functional performance should be interpreted cautiously.

More specifically, the functional differences across body composition phenotypes varied by functional domain. The high-adiposity–high-muscle phenotype showed the highest grip strength, whereas the low-adiposity phenotype showed better performance in one-leg stance with eyes closed and the 30-s chair stand. No significant difference was observed in 2-min high-knee stepping in place. These findings are generally consistent with previous studies showing that body composition is closely associated with physical function in older adults (26, 27). Greater muscle reserve may be associated with higher strength output, whereas greater fat accumulation may be related to greater mechanical burden and poorer balance-related or lower-limb functional performance (27, 28). Notably, the high-adiposity–high-muscle phenotype did not show uniformly poorer functional performance, suggesting that functional performance should not be interpreted in relation to adiposity alone, but in the context of the combined distribution of fat and muscle (29, 30). This finding further supports the value of identifying body composition phenotypes based on multiple indicators, rather than relying only on single measures such as BMI, which may not adequately capture functional heterogeneity among older adults (13, 14, 31). In addition, the observed phenotype-related differences in grip strength, balance, and chair-stand performance, but not in 2-min high-knee stepping, suggest that the relationship between body composition phenotypes and physical function may differ across functional domains (27, 32, 33).

These findings should be interpreted as phenotype-specific functional patterns rather than as a simple ranking of better or worse body composition profiles. No phenotype showed a uniformly favorable functional profile across all outcomes. Lower adiposity may correspond to lower mechanical burden during weight-bearing tasks such as chair stands and single-leg standing, whereas greater muscle reserve may be more closely related to maximal strength output such as grip strength. However, when higher muscle mass occurred together with higher fat accumulation, the observed strength advantage was not accompanied by better balance or chair-stand performance. This pattern suggests that the functional implications of body composition should be interpreted in relation to the combined distribution of fat and muscle, rather than adiposity or muscle mass alone (29, 30). Importantly, although several between-phenotype differences were statistically significant, the effect sizes were small, with partial η^2^ values of 0.019–0.022. Therefore, these phenotypes should not be treated as clinically established diagnostic categories for individual-level decision-making. Nevertheless, they may still be useful for describing population-level functional heterogeneity in community-dwelling older adults and for identifying subgroups with different functional strengths and vulnerabilities. Further longitudinal studies using clinically validated cut-off values are needed to determine whether membership in these phenotypes can predict clinically meaningful functional decline or adverse events over time.

In addition to body composition phenotypes, this study also found that upper-limb AI was nominally associated with sample-defined low balance performance, although this association did not remain significant after FDR correction. No significant association was observed for lower-limb AI. This suggests that the association between muscle asymmetry and functional performance may not be consistent across all outcomes, but may be more evident for specific outcomes such as balance or mobility (34, 35). One possible explanation is that asymmetrical upper-limb muscle distribution may reflect broader impairment in postural control. Because upper-limb function is also involved in balance regulation, such asymmetry may appear as a potential marker of reduced balance performance (34, 36). However, this outcome reflected general static balance performance rather than side-specific balance asymmetry because side-specific one-leg stance data were not retained. In addition, no significant association was observed in the linear regression analysis, whereas a nominal association was found in the logistic regression analysis. This apparent difference may reflect the fact that the logistic model examined a dichotomized, sample-defined low-performance outcome, whereas the linear model evaluated variation across the full continuous range of balance performance. However, because the logistic association was weak and did not remain significant after FDR correction, its clinical relevance remains uncertain and it should be regarded as an exploratory finding rather than robust evidence. No significant association was found for lower-limb AI. This may be because balance performance is related to multiple factors, including lower-limb strength, proprioception, and neural control. Therefore, structural bilateral differences based only on muscle mass may not be sufficient to characterize balance-related performance (37, 38).

Stratified analyses further showed nominal associations between upper-limb AI and risk of low balance performance in women and in older adults aged ≤75 years, and the extreme-group analysis showed a similar nominal pattern. However, these associations did not remain significant after FDR correction, and the interaction terms for sex and age group were not significant. These findings suggest that the association between upper-limb asymmetry and low balance performance may vary across populations, but should be interpreted cautiously. Women generally have lower overall muscle reserve, and therefore associations between bilateral asymmetry and functional performance may be more apparent when side-to-side differences occur (39, 40). In addition, the association pattern related to this type of asymmetry may differ across age groups and may be more detectable in relatively younger older adults with low functional performance (38). Nevertheless, the subgroup and extreme-group findings should be regarded as exploratory and require further validation. Overall, this study revealed potential characteristics of functional heterogeneity in community-dwelling older adults from two perspectives, namely body composition phenotypes and muscle asymmetry. These findings may provide a new perspective for the early identification of older adults with relatively poorer functional performance and may help inform the development of more targeted health management and intervention strategies.

## 5 Conclusion

In community-dwelling older adults, three distinct body composition phenotypes were identified, and significant differences in several functional performance indicators were observed across phenotypes. Limb muscle mass asymmetry was not robustly associated with functional performance after correction for multiple comparisons. Upper-limb AI showed only a nominal association with low one-leg stance performance, and this finding should be interpreted cautiously. These results suggest that body composition phenotypes may provide useful information for understanding functional heterogeneity and identifying older adults with relatively poorer functional performance. Given the cross-sectional design, these findings should be interpreted as associations and group-level functional patterns rather than evidence of causal effects.

## 6 Limitations

This study has several limitations. First, because of the cross-sectional design, causal relationships among body composition phenotypes, muscle asymmetry, and functional performance cannot be inferred. The regression models were adjusted only for age and sex. Therefore, residual confounding by BMI, physical activity, comorbidities, medication use, fall history, limb dominance, nutritional status, lifestyle factors, and overall functional health status cannot be excluded. Body composition was assessed using bioelectrical impedance analysis, which may be influenced by hydration status, recent food intake, physical activity, and device-specific algorithms. Although standardized procedures were used, not all participants were measured under strictly fasting conditions. This may have introduced measurement error and potential misclassification of body composition phenotypes.

Second, low functional performance was defined using the sample-specific first quartile rather than clinically established cut-off values. Therefore, the logistic regression results should be interpreted as relatively low performance within this sample, rather than as clinically diagnosed balance dysfunction or functional impairment. Although FDR correction was applied, multiple outcomes and subgroup analyses were examined, so chance findings cannot be completely excluded. Although participants were recruited from both urban and rural communities, all were from a single geographic region in Anhui Province. Differences in socioeconomic status, lifestyle, healthcare access, and body composition patterns across regions may limit the generalizability of the findings. Future multicenter studies are needed to validate these findings in more diverse older adult populations. Finally, physical function assessment mainly focused on grip strength, balance, and simple physical fitness tests. The asymmetry index was based on structural measurements of limb muscle mass and did not include side-specific muscle strength, dynamic motor performance, or side-to-side balance differences. Because only one final recorded value for the one-leg stance test was retained, weaker-side performance and bilateral balance differences could not be analyzed. This may have reduced sensitivity to detect functional consequences of asymmetry.

## Data Availability

The original contributions presented in this study are included in this article/Supplementary material, further inquiries can be directed to the corresponding author.
