# Supplementary material for: Associations between body composition phenotypes and physical function in community-dwelling older adults: an exploratory analysis of limb muscle mass asymmetry
Source: Front Med (Lausanne). 2026 Jul 17;13:1862786. doi: 10.3389/fmed.2026.1862786 (PMC13423943; doi:10.3389/fmed.2026.1862786)
Supplement: Supplementary file 1 [file Table_1.docx]

**Table S1. Baseline characteristics of the study participants.**

**Table S2. Unadjusted functional outcomes across body composition phenotypes**

**Table S3. Linear regression analysis of limb muscle mass asymmetry indices and continuous functional performance outcomes**

**Table S4. Interaction between limb muscle mass asymmetry indices and body composition phenotypes for continuous functional performance outcomes**

**Table S5. Extreme-value analysis of limb asymmetry and low balance performance**

**Table S1. Baseline characteristics of the study participants.**

| **Variable** | **Total sample (N = 600)** |
| --- | --- |
| **Demographic characteristics** |  |
| Sex, n (%) |  |
| 0 | 369 (61.5%) |
| 1 | 231 (38.5%) |
| Age, years | 67.78 ± 5.00 |
| Residence (urban/rural), n (%) |  |
| 0 | 479 (79.8%) |
| 1 | 121 (20.2%) |
| **Anthropometric measures** |  |
| Height, cm | 161.51 ± 7.66 |
| Weight, kg | 63.88 ± 10.15 |
| BMI, kg/m² | 24.43 ± 3.11 |
| Waist circumference, cm | 86.02 ± 11.01 |
| **Body composition** |  |
| Body fat percentage (%) | 30.67 ± 6.71 |
| Fat mass, kg | 19.72 ± 5.59 |
| Fat-free mass, kg | 44.23 ± 7.93 |
| Total muscle mass, kg | 41.52 ± 7.47 |
| Trunk fat mass, kg | 11.79 ± 3.33 |
| Left lower limb muscle mass, kg | 7.38 ± 1.35 |
| Right lower limb muscle mass, kg | 7.37 ± 1.34 |
| **Functional performance** |  |
| 30-s chair stand, repetitions | 16.77 ± 4.66 |
| 2-min step test, steps | 95.06 ± 30.61 |
| Handgrip strength, kg | 23.56 ± 7.24 |
| One-leg standing (eyes closed), s | 8.73 ± 6.45 |

**Table S2. Unadjusted functional outcomes across body composition phenotypes**

| **Outcome** | **C1(n = 164)** | **C2(n = 332)** | **C3(n = 104)** |
| --- | --- | --- | --- |
| 30-s chair stand (reps) | 17.41 ± 4.92 | 16.80 ± 4.58 | 15.67 ± 4.33 |
| One-leg stance with eyes closed (s) | 10.00 ± 7.80 | 8.02 ± 5.69 | 8.97 ± 6.14 |
| Grip strength (kg) | 22.30 ± 6.61 | 23.74 ± 7.45 | 24.99 ± 7.24 |
| 2-min high-knee stepping in place (steps) | 94.20 ± 25.20 | 94.62 ± 31.92 | 97.81 ± 34.02 |

Note. Values are presented as mean ± SD. C1, low-adiposity phenotype; C2, balanced phenotype; C3, high-adiposity–high-muscle phenotype.

**Table S3. Linear regression analysis of limb muscle mass asymmetry indices and continuous functional performance outcomes**

| **Predictor** | **Outcome** | **β** | **95% CI** | **P value** | **FDR-adjusted P** |
| --- | --- | --- | --- | --- | --- |
| Upper limb AI | 30-s chair stand (reps) | -0.010 | -0.088, 0.069 | 0.805 | 0.920 |
|  | One-leg stance with eyes closed (s) | -0.039 | -0.118, 0.041 | 0.339 | 0.905 |
|  | Grip strength (kg) | -0.002 | -0.066, 0.062 | 0.947 | 0.947 |
|  | 2-min high-knee stepping in place (steps) | -0.015 | -0.095, 0.066 | 0.720 | 0.920 |
| Lower limb AI | 30-s chair stand (reps) | -0.068 | -0.146, 0.010 | 0.087 | 0.697 |
|  | One-leg stance with eyes closed (s) | -0.017 | -0.096, 0.062 | 0.674 | 0.920 |
|  | Grip strength (kg) | 0.019 | -0.044, 0.082 | 0.557 | 0.920 |
|  | 2-min high-knee stepping in place (steps) | -0.045 | -0.124, 0.035 | 0.269 | 0.905 |

Note.Values are standardized β coefficients with 95% confidence intervals. Models were adjusted for age and sex. FDR-adjusted P values were calculated using the Benjamini–Hochberg procedure.

**Table S4. Interaction between limb muscle mass asymmetry indices and body composition phenotypes for continuous functional performance outcomes**

| **Predictor** | **Outcome** | **Interaction F** | **Interaction P** | **FDR-adjusted P** |
| --- | --- | --- | --- | --- |
| Upper limb AI | 30-s chair stand (reps) | 0.804 | 0.448 | 0.717 |
|  | One-leg stance with eyes closed (s) | 2.662 | 0.071 | 0.347 |
|  | Grip strength (kg) | 0.121 | 0.886 | 0.982 |
|  | 2-min high-knee stepping in place (steps) | 2.456 | 0.087 | 0.347 |
| Lower limb AI | 30-s chair stand (reps) | 0.019 | 0.982 | 0.982 |
|  | One-leg stance with eyes closed (s) | 0.544 | 0.581 | 0.774 |
|  | Grip strength (kg) | 1.243 | 0.289 | 0.704 |
|  | 2-min high-knee stepping in place (steps) | 1.047 | 0.352 | 0.704 |

Note.Interaction terms were tested in linear regression models adjusted for age and sex. Body composition phenotype was treated as a categorical moderator. FDR-adjusted P values were calculated using the Benjamini–Hochberg procedure.

**Table S5. Extreme-value analysis of limb asymmetry and low balance performance**

| **Predictor** | **OR (95% CI)** | **p value** | **FDR-adjusted P** |
| --- | --- | --- | --- |
| Upper limb AI:(highest 10% vs lowest 10%) | 1.870 (1.021, 3.426) | 0.043 | 0.085 |
| Lower limb AI:(highest 10% vs lowest 10%) | 1.188 (0.599, 2.357) | 0.621 | 0.621 |

Note:Low balance performance was defined as one-leg stance with eyes closed ≤5.0 s. Extreme asymmetry was defined as the highest 10% versus the lowest 10% of the AI distribution. Models were adjusted for age and sex. FDR-adjusted P values were calculated using the Benjamini–Hochberg method.
